# Healing dynamics and surgery in breast cancer: rethinking a timeless challenge in light of advancing therapies and technologies

**DOI:** 10.1186/s12967-026-07729-7

**Published:** 2026-01-24

**Authors:** Stefano Vinci, Roberta Biciuffi, Elena Barbieri, Giosuè Annibalini, Mauro De Santi, Daniela Lucini, Daniela Bosisio, Carolina Gaudenzi, Nicoletta Gagliano, Romano Demicheli, Elia Biganzoli, Elda Tagliabue, Francesca Bianchi

**Affiliations:** 1 Department of Biomedical Science for Health, University of Milan, Milan, Italy; 2 Department of Biomedical and Clinical Sciences, Medical Statistics Unit, “Luigi Sacco” University Hospital, University of Milan, Milan, 20157 Italy; 3 Department of Biomolecular Sciences, University of Urbino Carlo Bo, Urbino, Italy; 4 BIOMETRA Department, University of Milan, Milan, Italy; 5 Exercise Medicine Unit, IRCCS Istituto Auxologico Italiano, Milan, Italy; 6 Department of Molecular and Translational Medicine, University of Brescia, Brescia, Italy; 7 Data Science Research Center, University of Milan, Milan, Italy; 8 Department of Experimental Oncology, Fondazione IRCCS Istituto Nazionale dei Tumori, Milan, Italy; 9 Laboratorio Morfologia Umana Applicata, IRCCS Policlinico San Donato, Milan, Italy

**Keywords:** Breast cancer, Wound healing, Surgery, Surgical techniques, Histology, Tumor microenvironment, Radiomics

## Abstract

**Background:**

During the last century, surgery became the primary approach for treating breast cancer. However, the introduction of new surgical techniques, increasingly less invasive and more conservative, did not always provide consistent benefits in inhibiting tumor progression.

**Main body:**

Inflammatory mechanisms involved in wound repair of surgical damage play a significant role in the progression of cancer. In this review we focused on the healing-induced inflammation processes that can favor residual tumor cells and on the latest current methods, which improve the rate of total tumor excision during the first operation or contribute to diminish host systemic inflammation boosting immune system activation in breast cancer patients.

**Conclusions:**

Surgery is still a crucial area of breast cancer research that attempts to understand how it might affect breast cancer progression and, ultimately, survival outcomes.

## Introduction

Since the end of 19th century, after significant advancements in anesthesia and antisepsis, surgery became the primary approach for treating breast cancer (BC) [1]. However, it became evident early on that surgical removal of breast carcinoma did not consistently confer benefits in terms of inhibiting tumor progression.

To explain why radical mastectomy -initially used as standard surgery therapy-, involving the removal of the entire breast, overlying skin, underlying muscles, and most lymph nodes, didn’t cure BC patients, Fisher’s hypothesis was considered. Fisher proposed that cancer spreads through the bloodstream even before clinical detection, influenced by tumor-host interactions [2]. Pillar studies conducted by Dr. Bonadonna that demonstrated beneficial results from adjuvant systemic therapies even in early BCs bolstered the systemic nature of the disease from the early phases [3, 4]. If disseminated tumor cells exist at the time of surgery, radical tumor removal may promote their expansion. The wound healing process, characterized by the release of various cytokines and growth factors [5], may establish conditions that are permissive to the survival and proliferation of residual tumor cells, thereby facilitating the growth of pre-existing micro-metastases and enhancing their cancer stem cell characteristics. This model helps explain the elevated recurrence rate in patients with aggressive BCs within the first 2 to 3 years post-surgery [6, 7] and highlights the specific benefit of adjuvant therapy in reducing early relapse rates.

To comprehend how surgical techniques may influence the progression of the disease and, ultimately, survival outcomes remain an important challenge.

Therefore, a deeper understanding of the wound-healing effects on the microenvironment of primary tumor and me-tastatic foci is crucial. Many studies have demonstrated that tumor progression is characterized by alternating phases of cell quiescence and growth, enabling its adaptation to host changing conditions [8]. Surgical removal of the primary tumor —a procedure that induces substantial local and systemic inflammation— can disrupt this state of balance, triggering the awakening of dormant tumor cells that were already disseminated at the time of primary tumor excision [9, 10].

In this review, we examine how wound healing can support progression of residual tumor cells at both local and distant sites upon surgery, the connection between postoperative wound healing and the outgrowth of residual tumor cells, with particular emphasis on how healing-associated inflammation influences the anti-tumor immune response.

In addition, we go through the latest current methods, considering both surgical, pharmacological and life-style approaches, which improve the rate of total tumor excision during the first surgery or contribute to diminish host systemic inflammation affecting immune system activation in BC patients.

While inflammatory and immune mechanisms underlying surgery-induced tumor promotion are discussed in detail, their clinical relevance ultimately converges on a limited number of actionable perioperative strategies, including surgical de-escalation when appropriate, modulation of perioperative inflammation, and optimization of patient systemic conditions before surgery.

## Healing-induced inflammation

The risk of distant recurrence after surgical removal of primary tumors peaks at around 2–3 years with higher rate for BCs presenting the most aggressive subtype, i.e., HER2-positive and Triple-Negative (TN), implying that surgery and factors released during the healing process impact residual cancer cells and, in a special way, those with aggressive molecular characteristics [11]. Wound healing’s more pronounced impact on promoting relapse in TN and HER2 tumors may be attributed to enhanced communication between tumor cells that are already disseminated at the diagnosis and the factors involved in the healing process.

These findings further support the close link between wound repair mechanisms following surgical removal and the processes that tumor exploit to progress. Wound healing mechanisms involve inflammatory mediators [12], growth factors, cell interactions [13], and processes like epithelialization [14], fibroplasia, angiogenesis, wound contraction [15], and remodeling [16]. Pro-inflammatory cytokines induced by wound healing push tumor cells toward a cancer stem cell (CSC) phenotype, contributing to local and systemic recurrence. Indeed, IL-1β, IL-6, activate the NF-κB/STAT3 signaling pathway, leading to CSC expansion, TGF-β promotes Epithelial-Mesenchymal Transition (EMT), a process that endows tumor cells with stem-like and invasive properties, facilitating metastasis, and, collectively, prolonged inflammatory signaling can reprogram differentiated tumor cells into stem-like cells [17]. Modifications induced by the tumor in the neighboring microenvironment play a crucial role in mediating tumor progression through the wound-healing process. Observations suggest that healing factors are influenced by surgical damage and the reactive stroma resulting from interactions between neoplastic cells and the surrounding microenvironment [18] that extend even further away from the primary tumor [19].

Molecular factors, such as IL-6, G-CSF, MCP-1, and Osteopontin (OPN), are enriched in wound fluid from invasive BC patients, independent of surgery extent. Higher levels of OPN in drainage from TN tumor surgery may explain the high recurrence rate of this BC subtype [20]. Indeed, OPN enrichment in the tumor microenvironment (TME) contributes in orchestrating an immunosuppressive metastatic niche through the recruitment and functional reprogramming of myeloid-derived suppressor cells (MDSCs), which inhibit anti-tumor immune responses and promote the survival and outgrowth of disseminated tumor cells. Furthermore, specifically in TNBC, OPN activates the PI3K/AKT/mTOR signaling pathway, enhancing tumor cell proliferation, angiogenesis, and resistance to ferroptosis via GPX4-mediated lipid peroxidation suppression [21].

These findings indicate that tumors with more aggressive characteristics may modify the surrounding environment such that molecules released by this altered tissue after surgery promote the growth of any local residual tumor cells or even distant cells as well as through factors released into circulation [18, 22].

## Impact of surgery on the anticancer immune response

**Fig. 1:**
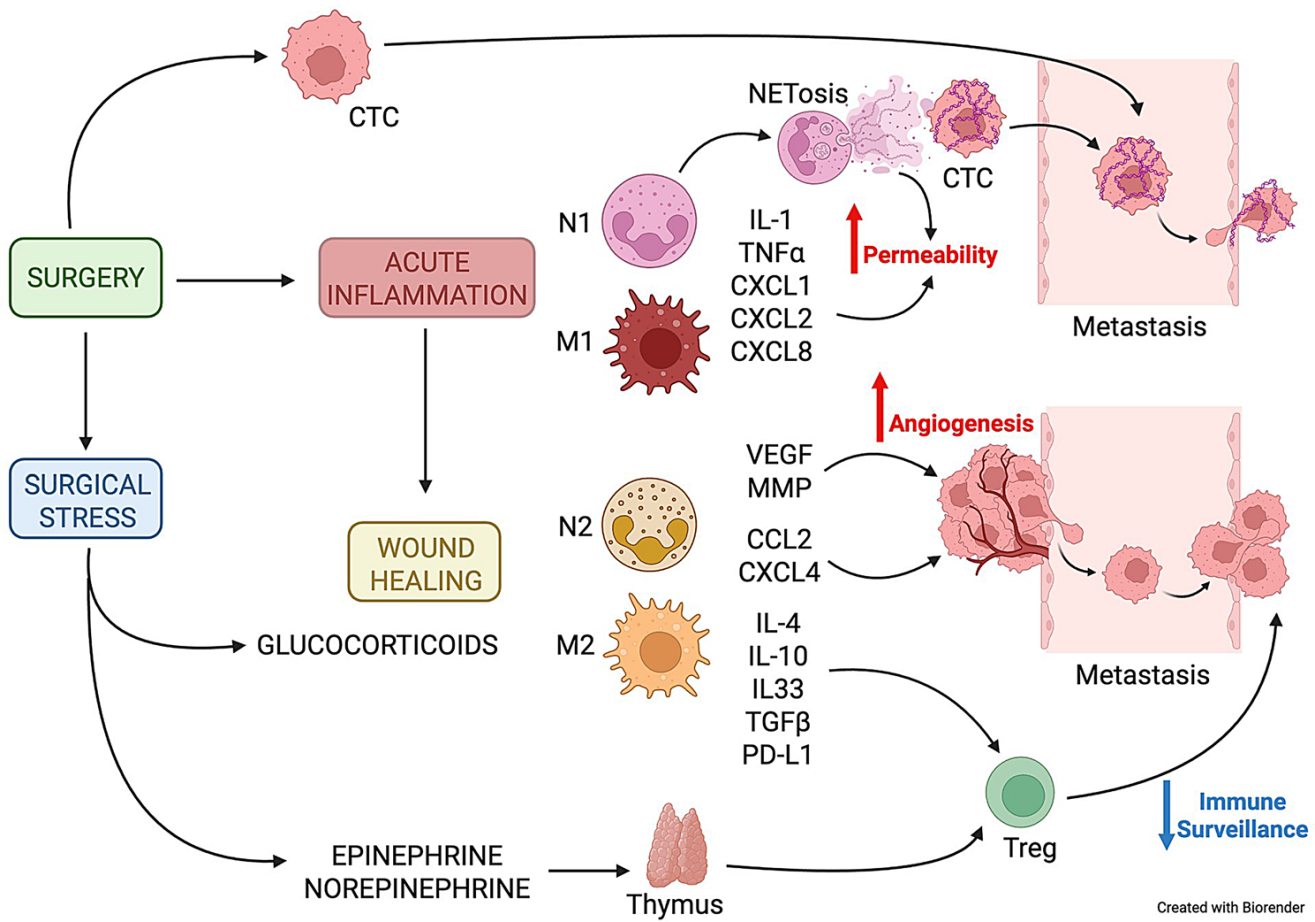
Mechanisms linking surgery-induced inflammation to cancer metastasis. This Figure depicts the complex wound-healing response initiated by surgical intervention leading to both surgical stress and acute inflammation

Tissue damage induced by surgery activates inflammation, which leads to wound healing through an ordered sequence of events consisting of acute inflammation, tissue repair, and tissue remodeling. Inflammation is tightly intertwined with the innate immune response since innate immune cells grant the order and timing of the whole inflammatory process. Thus, the physiological phenomenon of post-surgery wound healing may impact cancer growth and metastasis, exploiting innate immune cells (Fig. 1). Post-surgery acute inflammation is often sterile, i.e. triggered by endogenous stress signals released by damaged tissues [6, 23], but may also depend on undesired pathogen colonization of the wound. In either case, damaged cells and resident innate immune cells release inflammatory mediators, including cytokines and chemokines such as IL-1α/β, TNFα, and CXCL1/2/8, which activate the endothelium to recruit more immune cells, with the overarching aims to prevent infection, eliminate damaged tissues, and trigger the adaptive immune response. Indeed, the cytokine cocktail of acute inflammation induces innate immune cells, mainly macrophages and neutrophils, to acquire a pro-inflammatory phenotype known as type 1 phenotype (M1 and N1, respectively) characterized by increased inflammatory cytokine production, phagocytic activity and antigen-presenting capability. Despite this phase is generally not rewarded as cancer promoting, a relevant pro-metastatic role is emerging for NETosis, a defense mechanism characteristic of N1 neutrophils [6, 24, 25]. To capture microbes, N1 cells release fishnet-like structures composed of chromatin and lytic enzymes (such as elastase and myeloperoxidase) called neutrophils extracellular traps (NETs), which were originally believed to represent a form of programmed cell death and thus named after NETosis [26]. Proteolytic enzymes vehiculated by NETs may favor local invasion of cancer cells by degrading the extracellular matrix (ECM) and loosening the basement membrane and tight junctions of capillary walls [27, 28]. In addition, NETs were shown to wrap up circulating tumor cells together with platelets and product of the coagulation cascade activated by surgery, thus protecting tumor cells from the shearing force of blood flow as well as from the recognition and subsequent attack of cytotoxic immune cells such as Natural Killer cells and Cytotoxic CD8 + T lymphocytes [29, 30]. Such aggregates also display an increased probability of stopping into capillaries because of their size and stickiness, thus favoring the establishment of metastatic foci [31]. It is worth recalling here that during acute inflammation capillaries are permeabilized and made sticky by inflammatory mediators to allow physiological leukocyte extravasation, which further increases the chances of metastases. Circulating NETs are in the limelight as diagnostic and prognostic markers: indeed, NET levels are increased in the blood of patients with different cancer types [32], including BC, and may correlate with poor survival [25].

Tissue repair begins when innate immune cells switch from the pro-inflammatory type 1 phenotype to the anti-inflammatory type 2 phenotype (M2 and N2) and is characterized by the release of growth factors engaging the progenitor function of endothelial and tissue cells to replace cells that were lost with surgery or destroyed by acute inflammation [33]. These functions, which are key to wound healing, can also promote tumor progression [34, 35]. Indeed, tumor-associated macrophages (TAM) and neutrophils (TAN) display an M2 and N2 phenotype, respectively [36, 37], and a low ratio between M1 (pro-inflammatory phenotype) and M2 cells correlates with poor prognosis in various cancer types [38–40]. M2 cells release the potent pro-angiogenic factor Vascular Endothelial Growth Factor (VEGF), which increases vascular permeability and enhances recruitment of immunosuppressive cell populations such as T regulatory cells [41], and ECM remodeling enzymes such as matrix metalloproteinases (MMPs). In addition, M2 and N2 secrete chemotactic cytokines such as CCL2 and CXCL4 that recruit and differentiate innate immune cells and further amplify type 2 polarization [42–44]. Even more importantly, M2 are responsible for terminating the immune response to allow complete wound healing through the production of immunosuppressive molecules: in tumors, such physiological function is hijacked and suppresses anticancer immune responses. Key molecules include IL-4, IL-10 and IL-33 that exert a positive feedback loop on M2 and N2 polarization and TGFβ and PDL-1, supporting the differentiation and function of T regulatory cells (Treg), which impede innate and adaptive immune cell activation through antigen-specific (expression of CTLA-4 and CD25) and antigen-independent mechanisms (secretion of IL-10 and TGFβ) [45–47]. In recent years anti-angiogenic agents, alone or in combination with inhibitors of regulatory pathways, such as PD-1 and CTLA-4, showed promising results in the treatment of solid tumors by hindering the formation of an immunosuppressive TME [48]. A possible contribution to type 2 polarization of immune cells and increased tumor growth/dissemination after surgery also comes from the release of adrenal hormones. Surgical stress and pro-inflammatory cytokines produced at the site of inflammation activate the hypothalamic-pituitary axis, which induces the release of anti-inflammatory glucocorticoid hormones by the adrenal cortex as a negative feedback loop to control and limit excessive inflammation. Elevated glucocorticoid levels contribute to the type 2 switch and indirectly stimulate locally the growth of residual tumor cells and of metastases at distant sites as previously described [49, 50]. In addition to glucocorticoids, adrenal glands respond to the stimulation of the hypothalamic-pituitary axis also by releasing epinephrine (or adrenalin) and norepinephrine (or noradrenalin) that activate immune cells by binding to adrenergic receptors (AR), with β2-AR being the most expressed [51–53]. β2-AR signaling promotes M2 polarization and inhibits dendritic cell differentiation, antigen presentation and production of inflammatory cytokines, while enhancing IL-10 and IL-33 [54–58]. In the thymus, where T cells are matured and selected, β2-AR signaling promotes the apoptosis of thymocytes, thus reducing thymus cellularity and output of T cells [59], but it also impairs T cell function by reducing the autocrine production of IL-2 and by promoting the immunosuppressive properties of Tregs, thus contributing to reduced tumor immune surveillance [60–62].

## Preclinical evidences on the breast cancer-promoting role of wound fluids

Several in vitro studies have been conducted to demonstrate the direct causal link between wound healing and BC progression. To assess the impact of healing factors on breast tumor cell lines, samples from postsurgical drainage have been collected and examined. Wound-healing fluids (WHF) have higher concentrations of cytokines, chemokines, and growth factors compared to normal serum, potentially influencing distant, disseminated, individual tumor cells [63]. Our pillar study first showed that postoperative wound fluids are able to stimulate in vitro the proliferation of BC cells [64]. Besides boosting proliferation, wound fluids collected postoperatively from BC patients have been shown to induce EMT in BC cells [65], to promote the drug resistance of primary BC cells to chemotherapy [63], to increase the expression of pro-metastatic drivers [66, 67], and to induce the enrichment of BC cells with stem-like phenotypes, via activation of STAT3 [68]. Enrichment analysis according to WHF treatment identified several gene sets differentially and significantly enriched in the WHF-treated cells characterized by over expression of genes related to response to wound, second messenger- and G protein-mediated-signaling, cytokine activity and locomotory characteristics [67].

All these studies well-established that WHF collected postoperatively from BC patients could enhance the aggressive properties of residual tumor cells after tumor removal. However, it remains under debate whether this effect is a nonspecific consequence of the general inflammatory response associated with tissue repair, or if the wound healing process specific to breast surgery exerts a direct and/or indirect influence on specific oncogenic pathways. The study by Krall and colleagues provides compelling pre-clinical evidence that the pro-tumorigenic effects observed post-surgery are not exclusive to BC surgeries but are a general consequence of surgical procedures and the associated wound healing process [69]. They suggested that surgery itself, regardless of the tumor’s location, can create a systemic environment conducive to tumor progression, particularly through the mobilization of inflammatory monocytes and the differentiation into TAM, which suppress T-cell–mediated tumor surveillance, thereby facilitating tumor growth. However, it has been shown that WHF derived from the breast where a tumor was present led to the formation of more invasive colonies than the WHF derived from the normal breast [70], suggesting that in addition to the cytokines and matrix metalloproteases associated with inflammation, wound fluid after BC removal may contain additional proteins from the TME. In agreement, our and other studies suggest that the composition of WHFs varies according to BC subtype [71] and also to surgical extent, indicating that both the TME and surgical context may modulate the pro-tumorigenic potential of these fluids. We demonstrated an increase in tumor cell proliferation, migration, and invasion in response to wound-healing processes, with highly aggressive BC cells being the most affected [18]. Moreover, although WHFs stimulate in vitro proliferation of BC cells of all intrinsic subtypes, the effect is more pronounced with drainage fluid from surgery on tumors of the same intrinsic subtype [22], suggesting a specificity due to the transformation of surrounding healthy tissue by the tumor itself in an opportunistic manner. Accordingly with our findings, the WHF of inflammatory BC (IBC) patients showed a significantly higher expression of various cytokines than in non-IBC patients and IBC-WHF exhibited a greater potential for inducing IBC cell invasion than non-IBC cells [72].

## Clinical evidence linking breast cancer surgery and healing

Two landmark randomized clinical trials established breast-conserving surgery (BCS) as a viable alternative to mastectomy for early BC. The NSABP B-06 trial compared radical mastectomy to lumpectomy with or without radiotherapy [73], while the Milan 1 trial evaluated radical mastectomy versus quadrantectomy with axillary lymph node dissection and radiotherapy [74]. Both these pillar trials found no differences in disease-free (DFS) or overall survival (OS) at 5 years, with consistent results at 20- and 25-year follow-ups [74–76]. Subsequent studies confirmed these findings, making BCS the preferred option for 60–70% of early BC cases. Notably, re-analysis of Milan 1 trials and population-based studies indicated a potential survival advantage for breast-conserving approaches over mastectomy for early aggressive BC subtype [77].

Invasive surgery leading to extensive healing may accelerate the progression of disseminated cells more than conservative surgical removal of the tumor. As mentioned before, patients undergoing mastectomy showed an enrichment of inflammatory mediators in postoperative drainage fluids derived from the breast surgical wound, supporting the notion that highly destructive surgeries increase inflammation at local site. In agreement with these objectives, the levels of growth factors and healing mediators present in drainage fluids upon surgery are significantly correlated with surgery damage [78].

Although sentinel lymph node biopsy (SLNB) remains often a standard procedure for staging BC, it represents a surgical intervention, even if minimally invasive and localized, and it may trigger substantial inflammation and wound-healing responses. Thus, the omission of SLNB in specific subgroups of BC patients has emerged as a viable approach, supported by robust data from clinical trials and retrospective studies, resulting in very recent new guidelines [79–81]. Evidence demonstrates that for women aged 65 and older with estrogen receptor-positive (ER+)/HER2-negative BC, axillary recurrence rates are exceptionally low when SLNB is omitted during BCS [82]. This has been confirmed by prospective studies like those conducted by Chung et al., which showed 3-year regional recurrence-free survival rates of 98.2%, suggesting SLNB omission is safe in this population [83]. Similarly, in the INT09/98 randomized clinical trial we investigated the impact of avoiding axillary surgery in patients with T1N0 BC [84, 85]. After a median follow-up of over 10 years, the study found no significant differences in OS between patients who underwent quadrantectomy with axillary lymph node dissection and those who had quadrantectomy without axillary dissection.

As a further confirm, data from the SOUND trial and the Choosing Wisely initiative have established that for patients with small, node-negative BC identified through preoperative imaging, omitting SLNB does not compromise DFS and OS [86, 87]. This conservative surgical approach not only minimizes surgical morbidity but also aligns with findings from the Z0011 and AMAROS trials, which highlighted low recurrence rates in BC patients with limited nodal metastases treated with SLNB alone instead of complete lymph nodes dissection [88, 89]. Consistently, in the INT09/98 trial, among patients who did not undergo axillary dissection, 9.0% experienced axillary lymph node recurrence, with a median time to recurrence of 30.0 months. Despite this, the absence of axillary surgery did not negatively impact OS, reinforcing the notion that axillary surgery may be unnecessary in certain patient populations. Moreover, retrospective analyses reveal that certain subgroups, such as older patients with small, low-grade tumors, benefit minimally from axillary staging in terms of survival outcomes. Despite this, disparities persist in the implementation of these guidelines, with socio-demographic factors influencing practice patterns. Together, these findings support the tailored omission of SLNB in carefully selected patients, potentially reducing overtreatment without adversely impacting oncological outcomes. Based on these findings, it is plausible that avoiding an additional, nevertheless minimal, surgical intervention, which inherently triggers inflammatory and wound-healing responses with systemic and local repercussions, may contribute to the observed non-inferiority effect.

In summary, evidence from landmark randomized trials and subsequent population-based studies consistently demonstrates that less invasive surgical approaches do not compromise outcomes in early BC. As a support, we reported the NSABP B-06 and Milan trials results, establishing the equivalence of BCS and mastectomy in terms of DFS and OS, and more recent trials such as SOUND to support the safe omission of additional axillary procedures in selected patients.

Another important issue to consider in healing management is the need for radicalization due to positive margins of excised tissue, that is, the presence of residual disease at the site of tumor removal. In this context, in patients with HER2-positive BC tumors, who underwent to radicalization, we observed an increase in proliferating tumor cells that were grown during healing processes consequent to first surgery, suggesting that healing factors are active in stimulating the proliferation of residual HER2-positive cells [64]. Besides radicalization, multiple surgeries can occur due to re-excisions for recurrence or contralateral tumors, as well as reconstructive procedures. Understanding the biological impact on BC prognosis of repeated surgical interventions is crucial. Our findings indicate that the biological consequences of BC removal differ from those of surgery alone, in agreement with pre-clinical data discussed above. Indeed, reconstructive procedures primarily promote the acceleration of microscopic metastatic growth, whereas surgical excision of a BC—the primary tumour, an ipsilateral breast tumour recurrence (IBTR), or a contralateral breast cancer (CBC)—results in both the disruption of tumour-induced homeostatic control over micrometastases and the stimulation of their outgrowth [90].

For these reasons, all technologies that can help improve the precision of radical BC excision during the first surgery can support and complement all the approaches described above to minimize residual disease and avoid a re-excision with consequent wound-healing effects.

Intraoperative margin assessment using fluorescence-guided systems has been shown to reduce re-excision rates in BCS. 3D-printed surgical guides (3DP-BSG) have been shown to enhance precision in tumor localization for BCS after Neoadjuvant Chemotherapy (NAC). These guides, based on MRI or other imaging modalities, reduce the potential for incomplete resections and support more precise surgeries. Similarly, radio-frequency identification (RFID) technology provides a non-radioactive alternative for localizing nonpalpable breast lesions during BCS, improving intraoperative accuracy and reducing unnecessary surgical extensions.

Thus, these new technologies offer promising approaches to enhancing surgical precision and reducing healing complications.

Collectively, these data suggest that limiting surgical extent, if appropriate, reduces tissue damage and the associated wound-healing response without adversely affecting prognosis, indirectly supporting the concept that surgery-induced inflammation may contribute to recurrence dynamics, particularly in aggressive disease.

Although the overall body of evidence supports a role for surgery- and healing-associated inflammation in BC outcomes, some results appear discordant across clinical and translational studies. These differences largely reflect patient selection (risk/subtype), extent of local or axillary procedures, type of local adjuvant treatment, and endpoint definition (local control vs. DFS, OS). To facilitate interpretation, representative examples of apparently divergent findings are summarized in Table 1.

**Table 1:** Heterogeneity of clinical evidence on surgery-associated inflammation and breast cancer outcomes

Clinical question	Studies or evidence	Contradictory findings	Critical interpretation
Extent of breast surgery (BCS vs. mastectomy)	Landmark randomized trials and long-term follow-up studies	Long-term equivalence in DFS and OS between BCS and mastectomy vs. a possible advantage of BCS in early aggressive subtypes	Apparent discrepancies reflect subtype-specific biology and differences in surgical tissue damage; More extensive surgery may induce a stronger inflammatory and wound-healing response, which could differentially affect aggressive tumors
Omission of SLNB / axillary surgery	Prospective trials and randomized studies in selected early BC	Safe omission of sentinel lymph node biopsy without compromising survival in selected patients vs. modest increases in axillary recurrence without impact on OS	Differences arise from the choice of endpoint: regional control vs. survival; Reduced surgical burden may lower inflammation while maintaining oncological safety in properly selected patients
Intraoperative radiotherapy (IORT) vs. EBRT	Randomized trials and long-term updates	Initial reports of equivalence followed by higher local recurrence rates with IORT in later analyses	Increased local tissue damage and scarring from combined surgery and radiation may amplify or prolong inflammatory responses, potentially offsetting benefits of immediate tumor cell eradication
Dietary and lifestyle interventions	Randomized dietary intervention studies	No significant reduction in recurrence in the overall population vs. reduced risk in highly adherent subgroups with metabolic improvement	The effectiveness of lifestyle interventions depends on the magnitude of biological change achieved; Benefits may emerge only when systemic inflammation and metabolic dysfunction are meaningfully improved
Exercise timing (post-surgery vs. pre-surgery)	Observational and interventional exercise studies	Established benefits in post-surgical settings vs. limited evidence in the pre-surgical or neoadjuvant phase	This reflects differences in evidence maturity rather than contradiction. Prehabilitation is a promising approach but remains underexplored mechanistically and clinically
NSAIDs and prognosis	Observational studies and ongoing randomized trials	Signals of reduced recurrence or mortality with perioperative NSAID use vs. heterogeneity and lack of definitive conclusions	Variability likely depends on drug choice, timing, dosage, tumor subtype, and host inflammatory status, supporting the need for controlled trials and biomarker-guided strategies

## Impact of healing management on cancer progression

Anti-inflammatory agents, such as Nonsteroidal Anti-Inflammatory Drugs (NSAIDs), have been explored post-surgery to mitigate the protumoral effects of the wound-healing process [91, 92]. The rationale for using NSAIDs is based on impairing PTGS-2, angiogenic activities of platelets, and Nitric Oxide (NO) production, impacting blood vessel proliferation [93–96]. Initial studies examining postoperative NSAID administration’s relationship with BC prognosis suggest a benefit in reducing BC mortality [97, 98]. However, variability in drug dosages and the link between differently reactive stroma of various BC subtypes warrant further research. Large-scale randomized controlled trials, such as the ADD-ASPIRIN clinical trial, aim to definitively establish NSAIDs’ impact on BC prognosis [99].

In addition to post surgery treatment, NSAIDs’ favorable safety profile has led to their administration in the intraoperative setting. The timing of drug administration, rather than dosage, may be a major determinant in reducing distant disease recurrence, especially early events [100–102]. As explained above, several angiogenic factors peak at the first day post-surgery during wound healing, supporting the significant benefit of NSAIDs in the perisurgical vs. postsurgical period. Moreover, observations in BC patients undergoing NAC suggest increased levels of inflammatory cytokines and chemokines in drainage samples, possibly due to Reactive Oxygen Species (ROS) release during tumor cell death. NSAIDs in the perisurgical period are proposed to counteract the increase of systemic levels of proinflammatory mediators. Additionally, NSAIDs lower the number of Circulating Tumor Cells (CTCs), proposing another mechanism to reduce the risk of metastasis [103].

This evidence coming from pharmacological managing of surgery period, additionally supports the role of surgery-induced inflammation in early relapses in BC, with WHF acting as a carrier of protumoral signals at the systemic level. Preclinical studies confirmed this hypothesized mechanism showing that, in experimental mouse models with murine mammary carcinoma cells, tumor resection increases the systemic inflammatory response and promotes the outgrowth of BC cells at distant sites. Perioperative treatment of mice with NSAIDs significantly impedes the surgery-induced progression of the tumor [69].

In addition to possible pharmacological treatments aimed at nonspecifically reducing the repair cascade or tailored toward specific inflammatory mediators proven to play a direct role in awakening dormant tumor cells, continuing to seek and develop surgical techniques that limit tissue damage as much as possible should remain the first approach to prevent or reduce the detrimental effects of repair mechanisms.

Cryoablation has emerged as a viable alternative to surgery for selected low-risk, early-stage BC patients. Studies show that ultrasound-guided cryoablation results in high patient satisfaction and effective tumor removal while minimizing invasive surgical interventions [100, 101]. Cryoablation may be particularly valuable for patients unable to tolerate surgery, offering an effective, minimally invasive alternative for selected patient populations, highlighting the importance of tailoring treatments to individual needs.

Preoperative partial breast irradiation (PBI), including single-dose MR-guided approaches, is currently of great interest as alternative to surgery in low-risk BC patients, due to its potential in inducing pathologic complete response (pCR) in such patients [102, 103]. Moreover, even without pCR, the tumor shrinkage upon a single pre-operative PBI dose can allow a more conservative surgery of healthy breast tissue followed by a reduced healing process.

Intraoperative radio (IORT) might reduce the risk of local recurrence by directly killing tumor cells and modifying the local microenvironment [104]. Studies on IORT, including the TARGIT-A trial, have initially demonstrated its equivalence to external beam radio (EBRT) in terms of local control and survival outcomes [105]. However, recent update highlighted concerns regarding higher rates of local recurrence associated with IORT, whether using electron-based or low-energy techniques [106]. Regarding toxicity, evidence suggests that IORT is associated with comparable or slightly lower toxicity rates, although there may be a higher risk of seroma requiring aspiration and fat necrosis. Moreover, IORT also induces histological tissue modifications, such as squamous metaplasia with cellular atypia, and triggers molecular changes linked to immune activation [107]. Thus, nevertheless the synergistic biologic effects of simultaneous surgery and radiation remain largely unclear, the non-superiority of IORT, if not an even worse performance, compared to EBRT might suggest that greater scarring due to radiation-induced tissue damage could offset the potential benefit of in situ tumor cell eradication by triggering a more intense or prolonged systemic inflammatory response (Table 1).

Finally, every lifestyle intervention that can diminish host systemic inflammation and boost immune system activation in BC patients may not only improve overall health but also, if the patient presents to surgery with an already better systemic immune and low-inflammatory condition, help limit the systemic response to wound healing, potentially reducing the adverse effects of inflammation. Among lifestyle factors, the capability of dietary interventions in modulating systemic inflammation is well attested, supporting immune competence, and potentially reducing recurrence risk in BC patients. Accumulating evidence suggests that a diet rich in plant-based, anti-inflammatory foods—such as the Mediterranean or macrobiotic dietary patterns, often together with physical exercise can influence biological pathways related to cancer progression and overall prognosis. The DIANA-5 trial, a randomized study evaluating the impact of a macro-Mediterranean diet on BC recurrence, found no significant difference in recurrence rates between the intervention group and the control group after 5 years [108]. However, in women with higher adherence to the dietary recommendations the Homeostasis Model Assessment of Insulin Resistance (HOMA-IR), a common measure of insulin resistance, showed a significant improvement and a lower risk of recurrence was observed, particularly in those with estrogen receptor-positive (ER+) tumors who were treated with tamoxifen. These findings suggest that only an effective comprehensive lifestyle intervention, able to significantly improve metabolic health, may have implications for cancer recurrence risk. Earlier work from the same group also emphasized the importance of individualized nutritional counseling for BC survivors, highlighting positive effects on weight control and inflammation markers [108]. In addition, more recent studies reinforce these findings, showing that anti-inflammatory diets may contribute to improved BC prognosis [109] and better cardiometabolic outcomes over time [110, 111] (Table 1).

Several evidences, specifically point to the benefits of physical exercise in the post-surgical context. However, there is still a major lack of knowledge on the role of exercise in pre-surgery/neoadjuvant settings. In this perspective, in BC patients, physical activity prescription before surgery can represent an inexpensive treatment to effectively extinguish surgery-induced inflammation, and prehabilitation interventions are assuming more importance going beyond the improvement in postoperative recovery and function [112–114]. Exercise may impact metabolic/immunological effects leading to the lowering of tumor proliferation activity levels as a marker of tumor distant spreading capability. Recently, we conducted a reanalysis of randomized clinical trials that investigated the effect of exercise on BC recurrence dynamics [115]. This analysis revealed a reduction in the risk of medium- and long-term recurrence, providing a foundation for further research into the role of exercise in pre- and post-operative settings. Aerobic exercise may reduce cancer incidence [111, 116]; it may positively affect many mechanisms implied in the pathogenesis and progression of BC; it may manage some side effects and improve patients’ tolerance to adjuvant therapies and quality of life [112–114]. Of particular interest is the capability of exercise to simultaneously act on mechanisms such as immunological, endocrine and autonomic controls. Exercise may interfere at the molecular level, inhibiting cancer cell proliferation and regulating cancer metabolism and the immune environment. Physical activity has so far been shown to reduce circulating levels of inflammatory markers and to modulate the production and activation of inflammatory cells through the instruction of hematopoietic progenitor cells; it may mitigate the immune aging stimulating the activity of NK cells, enhancing antigen presentation, reducing inflammation and preventing the accumulation of aging cells [114, 117]. In vitro studies with exercise-conditioned sera support that physical activity improving patient status, may influence tumor progression and the TME. For instance, we observed that exercise-conditioned human sera reduce proliferation capability and tumorigenic potential of BC cells, evaluated in a 3D culture model, confirming that systemic factors might modify tumorigenic potential [118]. Further, Baldelli et al. [119] found that sera conditioned by high-intensity exercise sessions can reduce the tumorigenic potential of cancer cells, providing additional insight into the role of physical activity in modifying cancer cell dynamics. More recently, we demonstrated that sera obtained after an home-based lifestyle intervention program reduced the tumorigenic potential of TN BC cells [120], suggesting that lifestyle interventions could reduce the systemic concentration of pro-tumorigenic factors and hence mitigate tumor progression. Furthermore, Bettariga et al. [121] reviewed the suppressive effects of exercise-conditioned serum on cancer cells, noting that the mode, volume, and intensity of exercise can significantly influence the outcome, potentially offering new therapeutic avenues for controlling surgery-driven cancer progression.

**Fig. 2:**
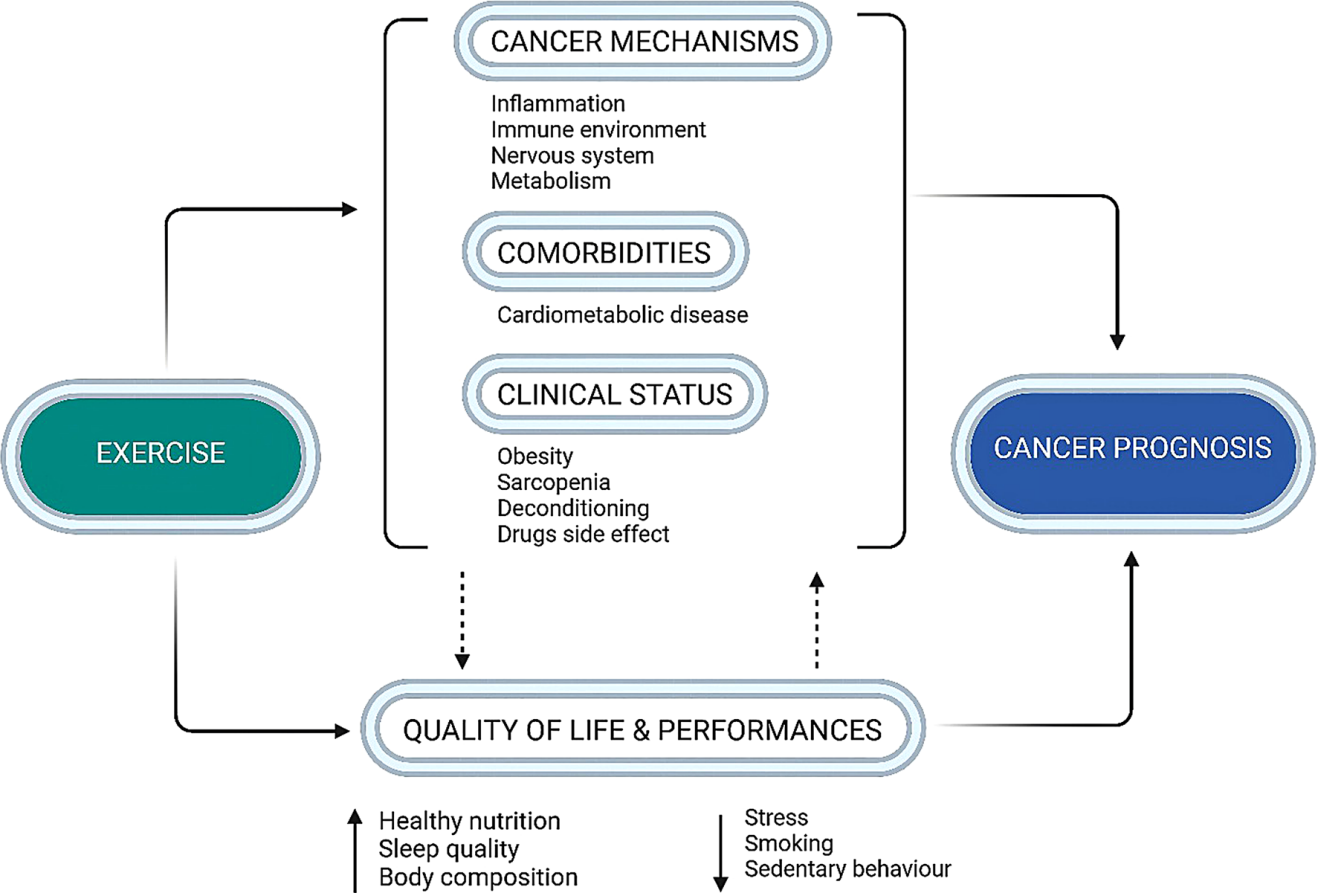
Multifactorial Impact of Exercise on Cancer Prognosis Exercise exerts direct effects on the mechanisms driving cancer progression, such as inflammation and metabolism, and contributes to the management of comorbidities and clinical status. It also enhances quality of life by improving sleep quality and body composition and promoting healthy behaviors, which further influence the biological mechanisms underlying cancer and contribute positively to prognosis. These effects emphasize the double impact of exercise on cancer prognosis, supporting exercise as a powerful, non-pharmacological intervention in oncological care

Another important effect of exercise is its capability to improve cardiorespiratory fitness [111, 116, 122], i.e. the efficiency with which oxygen is transported from the external environment into mitochondria in muscle cells, which is impaired in cancer patients due to many reasons ranging from physical deconditioning, increased age, treatments and specific effects of cancer which may affect the functional or structural integrity of oxygen cascade components, particularly at muscular level [122]. Cardiorespiratory fitness is also strictly dependent on autonomic nervous system (ANS) control which directly determines exercise execution [111, 122]; notably, there is a shred of growing evidence that ANS might play a pivotal role also in cancer pathogenesis and progression [123–127], particularly considering its relationship with the immune system [128, 129]. Efferent vagus nerve-mediated cholinergic signaling controls immune function and proinflammatory responses via the inflammatory reflex [128] inhibiting the inflammatory response [129]. Emerging data shows the existence of a bidirectional communication between nerve fibers and tumors they innervate and that excess activity in either the parasympathetic or sympathetic control might be associated with tumor growth and metastasis [124]. Moreover, we have to consider the important role of psychosocial stress as a possible facilitating factor in cancer disease and progression [124, 130], which may be explained by taking into account also the well-known negative modulation of immunological and autonomic controls due to stress [131]. Aerobic exercise, vice-versa, represents an important non-pharmacological strategy to improve ANS control also in cancer [127, 132, 133]. Exercise may be useful for BC management before and after surgery, also considering its indirect positive effects in reducing stress [122], optimizing body composition and cardiometabolic risk, reducing treatment side effects, and improving well-being and quality of life [112, 134, 135] (Fig. 2). Of paramount importance is that to get all the benefits that exercise may grant in BC patients, it needs to be prescribed, defining the precise goals to reach, considering the patient’s clinical condition, stage of the disease and patient preferences [136]. International guidelines well indicate the modality and volume of exercise required [134, 135, 137]; briefly, to improve cardiorespiratory fitness and BC prognosis, at least 150–300 min/week of aerobic exercise at moderate intensity is needed, alongside reducing as much as possible sedentary behavior and improving muscle strength. Exercise represents a promising strategy for improving BC management from pre-surgery to post-surgery interventions, capable of directly improving many mechanisms implied in cancer pathogenesis and progression (immunological, metabolic, autonomic controls), and of indirectly acting on factors release during healing which may favor cancer onset and dissemination. Nevertheless, despite growing evidence, the effects of exercise on inflammation and immune responses—both within the tumor microenvironment and systemically—remain largely unexplored, and there is a need for further investigation into the mechanisms by which exercise could mitigate inflammation and improve outcomes for cancer patients (Table 1).

## Post-surgical patient evaluation

Beyond perioperative interventions, a complementary and increasingly relevant perspective is the post-surgical, longitudinal, biological evaluation of the patient, aimed at capturing systemic inflammatory and stress responses triggered by tumor resection. In this context, biofluid-based omics (particularly serum/plasma metabolomics) offers minimally invasive, longitudinal readouts of host metabolic–immune adaptations during the early post-operative window, with potential implications also for personalized surveillance.

In BC, serum metabolomic profiling, particularly analyses performed in the post-surgical setting, can identify patterns associated with subsequent relapse risk and may therefore support risk stratification during follow-up [138, 139]. More broadly, perioperative metabolomics is being leveraged to characterize acute surgery-induced metabolic shifts in fluids such as plasma/urine, providing an additional framework for biomarker discovery linked to surgical stress and related-inflammation [140].

More recently, the post-operative assessment approaches have expanded toward multi-analyte circulating biomarker strategies (inflammatory mediators, adipokines, tissue damage or stress markers, immunosuppression-related signals, and other blood-based markers) to support precision perioperative management [141], as well as molecular residual disease approaches (e.g., ct DNA and CTC) that may refine early relapse detection in high-risk settings [142]. In parallel, digital info derived from wearables and mobile technologies are emerging as innovative tools to track recovery and patient’ physiology after surgery and could be integrated with omics fluids analysis to strengthen personalized long-term monitoring [143].

## Beyond wound healing: systemic factors in tumor recurrence

Beyond wound-healing–associated inflammation, additional clinical and molecular factors are recognized as contributors to BC recurrence following surgical intervention. As mentioned above, disruption of systemic and local homeostasis—particularly involving immune, endocrine, and metabolic pathways—can significantly modulate the post-operative TME [8, 10]. Chronic inflammatory comorbidities such as obesity, metabolic syndrome, diabetes, and autoimmune diseases establish a persistent pro-tumorigenic state, often characterized by elevated levels of IL-6, TNF-α, and other cytokines that can synergize with surgery-induced tissue damages in priming distant niches for metastatic colonization [144]. Furthermore, perioperative stress, including activation of the hypothalamic-pituitary-adrenal (HPA) axis and sympathetic nervous system, can drive the release of glucocorticoids and catecholamines, which impair anti-tumor immunity and promote M2-like polarization of macrophages and expansion of regulatory T cells. Commonly used anesthetics and analgesics may also exert immunomodulatory effects, potentially favoring or impairing tumor cell survival and dissemination.

These insights emphasize that BC recurrence is not solely dictated by the extent of tumor removal or the inflammatory response induced, but is the result of a complex interplay between tumor biology, host systemic conditions, and therapeutic context. Current challenges include the lack of reliable biomarkers to predict which patients are most vulnerable to surgery-induced recurrence and the need for tailored perioperative strategies that minimize detrimental host responses without impairing wound healing.

Future directions should focus on the integration of molecular profiling, stress-response modulation, and personalized pharmacological or behavioral interventions to mitigate the pro-metastatic impact of surgery and optimize long-term outcomes in BC patients. Since, as we mentioned, higher inflammation contributes to a higher risk of relapses, and could be helpful to implement lifestyle interventions, such as exercise or dietary recommendations, or treatments such as perioperative NSAIDs could be implemented to reduce systemic inflammation both before and after surgery, which could also prove useful to slow cancer progression before surgery.

## Conclusions

As surgery remains the most effective treatment for BC, understanding the inflammatory response during local wounding and its consequences on disease progression is crucial. Tissue damage due to cancer surgery provides a favorable niche for tumor recurrence, impacting pre-existing micro-metastases, the cancer stem cell population, and creating a ROS-rich environment, ultimately influencing patient outcomes. Evidence from in vivo and in vitro studies consistently demonstrates that wound-healing fluids, rich in cytokines, chemokines, and growth factors, can stimulate tumor cell proliferation, migration, and invasion, with the most pronounced effects observed in aggressive BC cells. However, life-style factors like physical activity may affect the tumor microenvironment potentially modulating cancer progression. Exercise-conditioned sera, for example, have shown the ability to alter BC cell behavior, and high-intensity exercise sessions have been linked to reduced tumorigenic potential. On the pharmacological front, anti-inflammatory agents such as NSAIDs offer promising post-surgical benefits by reducing inflammation and limiting the spread of tumor cells, with early studies suggesting a reduction in BC mortality when NSAIDs are used perioperatively. Additionally, emerging surgical techniques, such as cryoablation and IORT, have shown potential in minimizing tissue damage and enhancing therapeutic outcomes. Future studies are needed to prospectively evaluate the integration of perioperative strategies aimed at mitigating surgery-induced systemic inflammation and its pro-tumorigenic consequences. In particular, the combination of short-term anti-inflammatory pharmacological interventions, such as perioperative NSAID administration, of minimal-invasive surgical techniques, with structured prehabilitation programs based on physical exercise interventions and lifestyle modifications warrants dedicated investigation. These approaches may act synergistically by reducing inflammatory and stress-related responses while enhancing immune competence at the time of surgery. Further research should aimed to define optimal timing, patient selection, and intervention intensity, as well as to identify circulating or functional biomarkers that can guide personalized perioperative management for improving both the immediate and long-term outcomes for BC patients.

## Data Availability

Not applicable.

## Abbreviations

3DP-BSG: 3D-printed surgical guides
ANS: Autonomic nervous system
AR: Adrenergic receptors
BC: Breast cancer
BCS: Breast-conserving surgery
CBC: Contralateral breast cancer
CSC: Cancer stem cell
CTCs: Circulating tumor cells
CTLA-4: Cytotoxic T-lymphocyte antigen 4
CXCL: C-X-C motif chemokine ligand
DSF: Disease-free survival
EBRT: External beam radiotherapy
ECM: Extracellular matrix
EMT: Epithelial-mesenchymal transition
ER: Estrogen receptor
GPX4: Glutathione peroxidase 4
HER2: Human epidermal growth factor receptor 2
HOMA-IR: Homeostasis model assessment of insulin resistance
IBC: Inflammatory breast cancer
IBTR: Ipsilateral breast tumour recurrence
IL: Interleukin
IORT: Intraoperative radiotherapy
MCP-1: Monocyte chemoattractant protein 1
MMPs: Matrix metalloproteinases
MRI: Magnetic resonance imaging
NAC: Neoadjuvant chemotherapy
NETs: Neutrophils extracellular traps
NK: Natural killer
NO: Nitric oxide
NSAIDs: Nonsteroidal anti-inflammatory drugs
OPN: Osteopontin
OS: Overall survival
PBI: Preoperative partial breast irradiation
pCR: Pathologic complete response
PDL-1: Programmed death-ligand 1
RFID: Radio-frequency identification
ROS: Reactive oxygen species
SLNB: Sentinel lymph node biopsy
STAT3: Signal transducer and activator of transcription 3
TAM: Tumor-associated macrophages
TGF: Transforming growth factor
TME: Tumor microenvironment
TN: Triple-negative
TNF: Tumor necrosis factor
Treg: T regulatory cells
VEGF: Vascular endothelial growth factor
WHF: Wound-healing fluid
